# Pediatric Slow-Progressive, but Not Non-Progressive Cerebellar Ataxia Delays Intra-Limb Anticipatory Postural Adjustments in the Upper Arm

**DOI:** 10.3390/brainsci13040620

**Published:** 2023-04-05

**Authors:** Silvia Maria Marchese, Roberto Esposti, Veronica Farinelli, Claudia Ciaccio, Arianna De Laurentiis, Stefano D’Arrigo, Paolo Cavallari

**Affiliations:** 1 Human Physiology Section of the DePT, Università degli Studi di Milano, 20133 Milan, Italy; 2 Department of Pediatric Neuroscience, Fondazione IRCCS Istituto Neurologico “Carlo Besta”, 20133 Milan, Italy

**Keywords:** genetic ataxia, generalized cerebellar atrophy, cerebellar vermis hypoplasia, postural control, compensatory strategies

## Abstract

We recently investigated the role of the cerebellum during development, reporting that children with genetic slow-progressive ataxia (SlowP) show worse postural control during quiet stance and gait initiation compared to healthy children (H). Instead, children with genetic non-progressive ataxia (NonP) recalled the behavior of H. This may derive from compensatory networks, which are hindered by disease progression in SlowP while free to develop in NonP. In the aim of extending our findings to *intra-limb* postural control, we recorded, in 10 NonP, 10 SlowP and 10 H young patients, Anticipatory Postural Adjustments (APAs) in the proximal muscles of the upper-limb and preceding brisk index finger flexions. No significant differences in APA timing occurred between NonP and H, while APAs in SlowP were delayed. Indeed, the excitatory APA in Triceps Brachii was always present but significantly delayed with respect to both H and NonP. Moreover, the inhibitory APAs in the Biceps Brachii and Anterior Deltoid, which are normally followed by a late excitation, could not be detected in most SlowP children, as if inhibition was delayed to the extent where there was overlap with a late excitation. In conclusion, disease progression seems to be detrimental for *intra-limb* posture, supporting the idea that *inter-* and *intra-limb* postures seemingly share the same control mechanism.

## 1. Introduction

Every reaction force induced on the body by any voluntary motor action needs to be counteracted by anticipatory fixation chains spreading over several muscles [1,2,3]. These fixation chains are part of a specific feedforward motor program [3], which, together with feedback control systems, aims to stabilize the body posture. When movement concerns large masses (for example, the entire arm), Anticipatory Postural Adjustments (APAs) develop in several muscles belonging to the trunk and the other limbs (*inter-limb* APAs). Since these perturbations may even dislocate the Center of Mass (CoM), *inter-limb* APAs are also involved in stabilizing the whole-body equilibrium [1,2,4]. On the other side, when the movement involves distal segments, even of tiny mass (for example, a single finger), APAs also develop in the same limb in which the movement occurs (*intra-limb* APAs) so as to grant local equilibrium to the limb. In this regard, it is also very important to recall that *inter- and intra-limb* APAs show a strict correspondence in their behavior and temporal/spatial organization [5], such that they may be seen as two aspects of the same phenomenon.

Compared to *intra-limb* APAs, there has been greater investigation into *inter-limb* APAs, such as when performing movements of the whole upper limb [6,7] or of the lower limbs and the trunk [8,9]. Instead, the *intra-limb* APAs have been described for movement concerning the elbow [10,11,12], the wrist [13] or the fingers [14]. According to these last authors, a brisk index finger flexion, with the hand prone, discharges an upward reaction force on the metacarpophalangeal joint, which would produce flexion at the elbow and the shoulder. To counterbalance such flexion torques, a fixation chain develops comprising an anticipatory excitation in Triceps Brachii (TB) and Superior Trapezius (ST), coupled to an anticipatory inhibition in Biceps Brachii (BB) and Anterior Deltoid (AD). Interestingly enough, when flexing the finger with the hand supine, the joint torques push toward extension, and accordingly, Caronni and Cavallari observed that the pattern of excitatory and inhibitory actions reverted in sign.

Among the neural structures involved in motor control, it is worth recalling that the cerebellum plays a key role in generating feedforward muscle synergies [15,16] and in the timing of motor events [17,18,19,20]. In this regard, Diedrichsen [21] reported that in the cerebellum of adult patients, the *inter-limb* APAs during a bimanual unloading task are preserved in a pattern (excitation and inhibition), but are abnormally timed. Similarly, by using the index finger flexion paradigm in slow-progressive ataxic adults, Bruttini et al. [22] reported that the *intra-limb* APA pattern was preserved, while timing was always delayed. Moreover, we recently investigated *inter-limb* APAs in the lower limbs during gait initiation in children suffering from genetic disorders, which resulted in either a slowly progressive (SlowP) or a non-progressive (NonP) cerebellar ataxia [23]. Interestingly enough, a significant whole-body postural impairment was observed only in SlowP children, while postural control in NonP was not significantly different from that of the healthy population. This observation highlighted the importance of disease progression in determining the postural deficit. The same study also stressed that a correct ontogenesis of the APA control requires either a healthy cerebellum or the recruitment/development of a compensatory network able to vicariate the cerebellar function. Considering that a strict correspondence has been highlighted in the behavior and temporal/spatial organization of both *inter-* and *intra-limb* APAs [5], it is reasonable to expect that the observations collected in gait initiation might be replicated when APAs develop in the same limb in which a distal segment is voluntarily moved.

Therefore, the purpose of this paper is to verify whether *intra-limb* APAs are significantly impaired by disease progression; should this be true, the suggestion of Farinelli et al. [23] could be considered a general principle encompassing all APAs. In this aim, we applied the index finger flexion paradigm in young patients suffering from genetic slow-progressive (SlowP) and non-progressive (NonP) ataxia.

## 2. Materials and Methods

### 2.1. Sample

**Figure 1:**
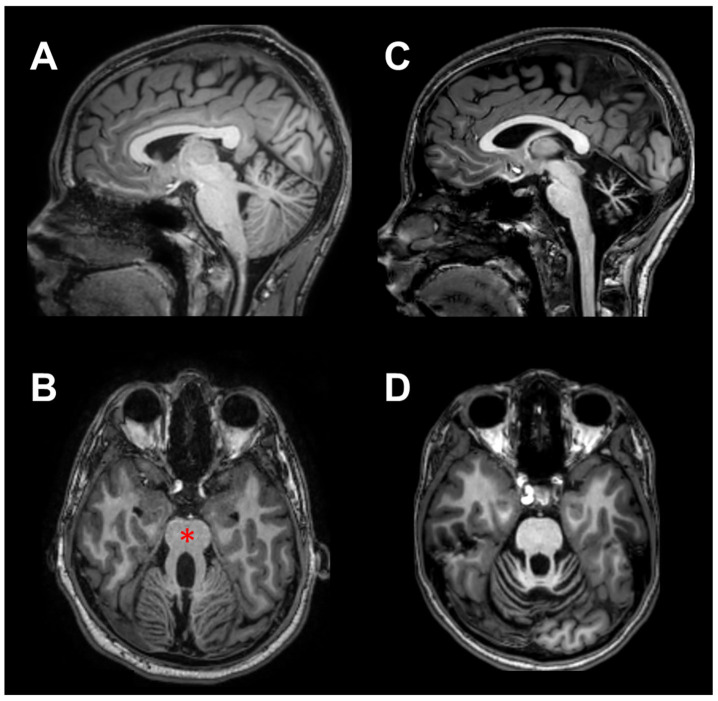
MRI scans of two representative ataxic children, in sagittal (**A**,**C**) and axial (**B**,**D**) views. In (**A**,**B**), scans of a child with non-progressive ataxia (NonP) show the typical vermian hypoplasia (“molar tooth” sign, red asterisk). In (**C**,**D**), scans of a child suffering from slow-progressive ataxia (SlowP) with evident generalized cerebellar atrophy are presented.

Nineteen children and a very young adult (20.7 years) with Pediatric Cerebellar Ataxia (PCA) of genetic origin were enrolled at the Istituto Neurologico “Carlo Besta” of Milan. Ten of them were affected by a non-progressive cerebellar pathology (NonP), classified as Joubert syndrome [24], showing a characteristic malformation known as the “molar tooth sign” on MRI (Figure 1B, red asterisk). This group comprised four females and six males of mean age 13.3 years ± 4.2 SD (range 8.8–20.7 years); it is important to note that one male subject was a young adult, although he was clinically followed by the pediatric department of the Istituto Neurologico “Carlo Besta”. For the sake of simplicity, this group has been referred to as NonP children throughout the manuscript. The other ten patients had clinical evidence of slow illness progression (SlowP) and showed radiological signs of generalized cerebellar atrophy (Figure 1C,D). This group included five females and five males of mean age 13.8 years ± 4.0 SD (ranging from 7.2 to 17.8 years). Due to the genetic origin of the disease, the duration of the illness was equal to the patients’ age. All patients followed standard physiokinesitherapy, except for two SlowP patients who followed psychomotricity training. Treatment started within two years of diagnosis and lasted 12.1 years ± 4.2 SD for the NonP group and 10.1 years ± 5.6 SD for the SlowP group. All cerebellar patients were able to understand and perform the motor task described below. Data collected from NonP and SlowP children were compared with those obtained in ten healthy children (H, six females and four males, mean age: 9.1 years ± 2.6 SD, range 6.0–14.0 years).

The experimental procedure was carried out in accordance with the standards of the Declaration of Helsinki. The Ethical Committee “Comitato Etico di Ateneo dell’Università degli Studi di Milano” approved the study and the written consent procedure (counsel 5/16). Although participants were free to quit the experiment at any time, all of them completed the experimental session.

### 2.2. Experimental Procedure

Participants were seated on a chair with both forearms on armrests, their backs supported, and both feet on the ground; they were asked to perform three sequences of 15 brisk flexions of the index finger at the metacarpophalangeal joint. In order to avoid any reaction time, each movement was performed at will after a beep (ready signal, repeated every 7 s).

Before each sequence, the subject lifted the preferred forearm from the arm rest and actively kept the upper arm vertical along the body with the elbow flexed at 90° and the hand prone, aligned with the horizontal forearm. In addition, the index finger was kept aligned with the hand, pointing forward, while all other fingers were hanging. The subject’s position was always visually controlled by the experimenter.

Starting from that position, the subject performed the sequence of 15 brisk index finger flexions, followed by slow returns to the pointing forward position. To avoid fatigue, the subject repositioned their forearm on the armrest after the sequence until they were ready to start a new sequence.

### 2.3. Data Collection and Analysis

The index finger movement was recorded at the metacarpophalangeal joint by a strain-gauge goniometer (mod. F35, Biometrics Ltd.^®^, Newport, United Kingdom) stuck on the skin with hypoallergenic tape. The angular signal was DC-amplified (P122, Grass Technologies^®^, West Warwick, RI, USA), and gain was calibrated before each experiment.

EMG signals were recorded by pairs of pre-gelled surface electrodes (H124SG, Kendall ARBO, Tyco Healthcare, Neustadt/Donau, Germany) placed on the muscle belly of the prime mover Flexor Digitorum Superficialis (FDS) and of the ipsilateral postural muscles Biceps Brachii, Triceps Brachii and Anterior Deltoid (BB, TB and AD, respectively). The inter-electrode distance was 24 mm, and electrode placement followed the SENIAM guidelines [25]. Recording selectivity was verified by checking that the activity from each recorded muscle during its phasic contraction was not contaminated by other muscular sources. EMG signals were amplified (IP511, Grass Technologies^®^, West Warwick, RI, USA) with a 1–20 k gain and a band-pass filter at 30–1000 Hz (2-pole high-pass and 4-pole low-pass) to minimize movement artifacts and high frequency noise.

Conditioned goniometric and EMG analog signals were then simultaneously sampled at 1 kHz, with an anti-aliasing 2-pole low-pass filter at 500 Hz and 12-bit resolution (A/D board PCI-6024E, National Instruments^®^, Austin, TX, USA).

Each EMG recording was digitally rectified. For each variable, the recorded traces (15 × 3) were time-aligned to the point at which finger flexion reached 15° with respect to its resting position (mean value from 1 to 0.1 s before the ready signal) and averaged. Such a choice was actually granted for time-alignment precision, as it was verified that at 15° flexion, the index finger was moving at more than 50% of its peak velocity. The resulting averaged trace extended from 2 s before the aligning point to 0.3 s after. All subsequent measurements were taken on the averaged traces.

The onset of index finger movement was automatically identified on the averaged goniometric trace. The mean signal level from 1 to 0.5 s before the ready signal (reference period) was subtracted from the trace, and then an algorithm searched for the moment in which the trace fell below −2 SD of the reference period and remained below that level for at least 50 ms. When the criterion was fulfilled, the algorithm searched backward for the time point at which the trace started to deviate from the reference period mean value. Movement amplitude and duration were measured as the amplitude and timing differences between the peak flexion of the index finger and the onset of its movement, respectively.

For each average EMG trace, the reference period was set from 1 to 0.5 s before the movement onset. The trace was integrated (time constant = 11 ms) and the mean reference level was subtracted from it, then the onset of an increment or of a decrement in EMG activity was identified by the above-described software algorithm, setting the threshold at +2 SD or −2 SD of the reference period signal, respectively. Considering that the decrements in tonic EMG activity of postural muscles are sustained by a central inhibitory command [26], we considered any increase or decrease in tonic EMG as excitatory/inhibitory APAs.

All measurements were visually validated to correct for possible failures of the automatic algorithm. Timings were expressed as latencies with respect to FDS onset, with negative values representing time advances.

### 2.4. Statistics

Statistics were applied to the following variables: APA latencies in the three postural muscles (BB, TB and AD), movement latency, duration and amplitude. For all tests, statistical significance was set at *p* < 0.05. Fisher’s exact test was used to compare the occurrences of BB and AD APAs in SlowP vs. H children. The Shapiro-Wilk test assessed the normal distribution of all extracted variables, except for BB and AD APA latencies in SlowP children, which included many missing values (see results). *T*-tests were applied to compare mean APA latencies in BB and AD of H vs. NonP children. One-way ANOVA and Tukey post-hoc tests were applied to compare mean TB APA latencies as well as mean movement latency, duration and amplitude. Levene’s tests were used to compare the variability of APA latencies in all muscles of H vs. NonP children as well as in the TB of H vs. SlowP children.

## 3. Results

### 3.1. EMG and Kinematics Recordings

**Figure 2:**
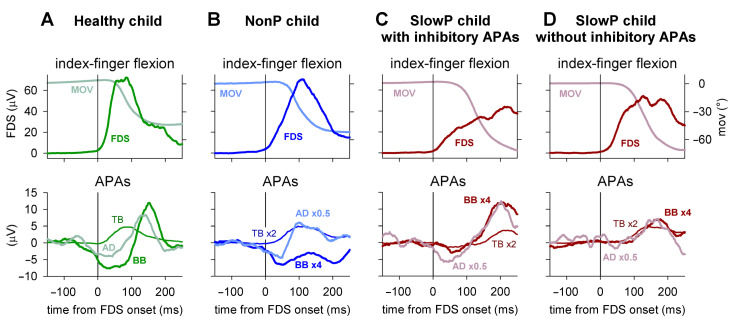
EMG and index finger kinematics obtained from four representative subjects. Averages of 45 movement trials, aligned to the onset of the prime moving muscle Flexor Digitorum Superficialis (FDS, solid vertical line). EMG was integrated for 40 ms. (**A**) One healthy child; the FDS onset was clearly preceded by inhibitory Anticipatory Postural Adjustments (APAs) in the Biceps Brachii (BB) and Anterior Deltoid (AD) and accompanied by an excitatory APA in the Triceps Brachii (TB). (**B**) One NonP child showing a behavior of recall comparable in pattern and timing to that of the healthy child. (**C**,**D**) Two SlowP children, one showing inhibitory APAs and one lacking them. Note that the APAs in TB were delayed in both SlowP children, which was also observed for the BB and AD APAs in the child in panel (**C**), while the APAs of the SlowP child shown in panel D were lacking, as if the delayed inhibition slipped under the following late excitation.

Typical EMG and kinematics recordings are illustrated in Figure 2. In the representative healthy child of panel A, the FDS EMG onset was clearly preceded by inhibitory APAs in BB and AD (latency of about --70 ms and about −50 ms, respectively), followed by a rebound excitation. Moreover, an excitatory APA developed in TB shortly after the FDS onset (about +10 ms). Thus, healthy children displayed APAs comparable in pattern and timing to those observed in healthy adults [5].

Among the ataxic patients, NonP children recalled the pattern and timing of the behavior of H children, as illustrated in the representative child in Panel B. In this case, the APA pattern was preserved (inhibition of BB and AD and excitation of TB), with latencies of about −20 ms for BB, −30 ms for AD and +10 ms for TB. In SlowP children, the excitatory APA in TB was always observed but largely delayed. In addition, the inhibitory APAs in BB and AD were delayed, but, in many cases, inhibition could not be identified. Panel C illustrates a child in which the complete APA pattern could be observed, but all APAs were clearly delayed, as they mainly developed after the FDS onset. Panel D shows another child in whom inhibitory APAs were undetectable and the excitation in TB had a long latency. In this case, it may be wondered whether inhibitory APAs were delayed to the extent whereby they slipped under the following rebound excitation.

### 3.2. Occurrences of Inhibitory APAs

Among SlowP children, only two showed inhibition in both BB and AD; two showed inhibition only in BB, while in the remaining children the inhibitions were completely undetectable. A Fisher’s exact test revealed that the occurrence of these APAs was significantly lower in SlowP vs. H children (*p* = 0.0054 for BB, *p* = 0.0004 for AD). Note that the time courses of both FDS recruitment and index finger kinematics were comparable in H and NonP children (Figure 2A,B), while they were somewhat longer in SlowP children (Figure 2C,D).

### 3.3. APA Latencies

**Figure 3:**
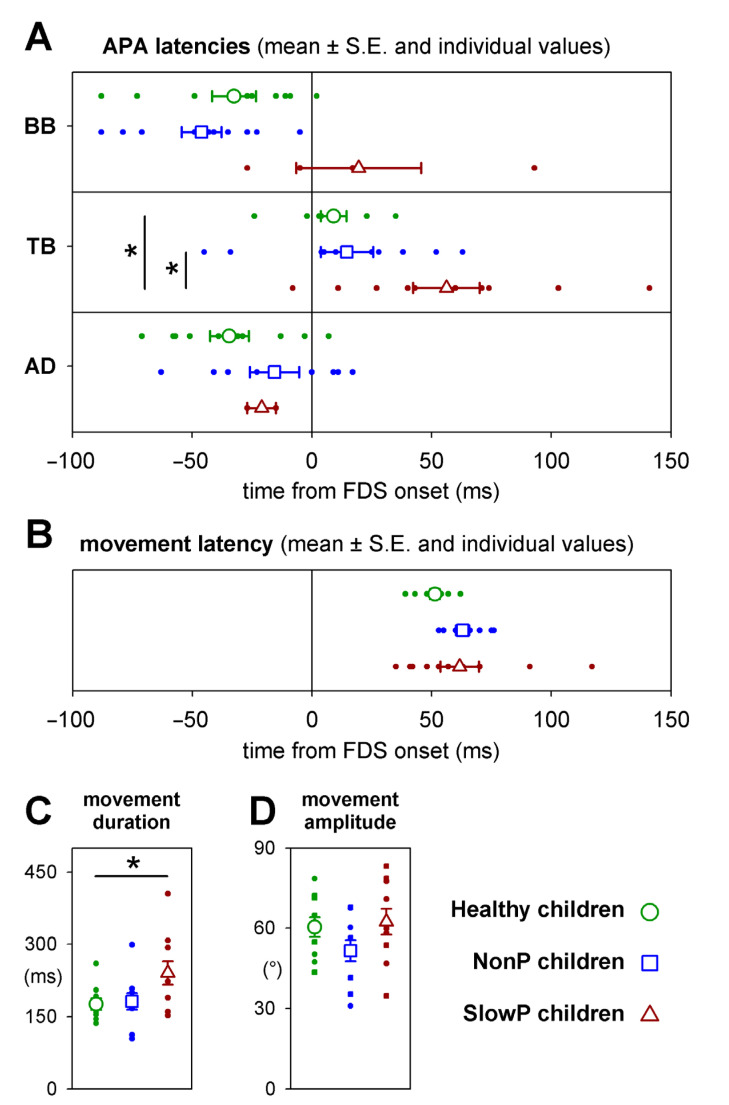
(**A**) Mean APA latencies in the three groups of patients ± SE (open symbols), together with individual values (filled circles), measured with respect to the FDS EMG onset. No significant timing differences occurred between NonP and Healthy children. In SlowP, the excitatory APA in TB was present in all 10 children but was significantly delayed with respect to both Healthy and NonP subjects, while inhibitory APAs could be detected in the BB in only 4 children and in the AD in only 2 children. (**B**) Latency, (**C**) duration and (**D**) amplitude of index finger movement in the three groups; same symbols as in panel (**A**). Latency and amplitude were comparable in the three groups, while movement duration was significantly longer in SlowP children. Significant differences are denoted by an asterisk (*).

Individual values of APA latencies, together with their means and standard errors, are plotted in Figure 3A. No significant (see below) time differences occurred in BB, TB and AD APAs between NonP and H children (blue squares vs. green circles, respectively), although two NonP children did not show inhibitory APAs in AD. With regard to SlowP children (brown symbols), the data about the inhibitory APAs in AD (two out of ten subjects) clearly do not represent the population’s behavior. A similar consideration should be drawn for BB (four out of ten subjects); however, note that APAs developing in this muscle were clearly delayed. For TB APAs, which were detected in all participants, it is apparent that they were delayed in SlowP children (about 50 ms), with respect to both H and NonP. It is important to note the larger variability of latencies in SlowP vs. H participants, especially in TB where there were no missing data.

With regard to the statistics about the data illustrated in Figure 3, APA latencies in BB and AD were compared only between H and NonP children because of the missing APAs in SlowP. T-tests and Levene’s test did not find any significant difference in mean latency (for BB t_18_ = 1.09, *p* = 0.29; for AD t_16_ = 1.46, *p* = 0.16) or variability (for BB F_1,18_ = 0.07, *p* = 0.79; for AD F_1,16_ = 0.47, *p* = 0.50). Instead, an ANOVA on TB APA latency encompassed all three groups. In this case, significant differences in mean latency were found (F_2,27_= 5.83_,_ *p* = 0.008) between SlowP and both NonP and H children (Tukey post-hoc *p* < 0.027); NonP were instead not different from H (Tukey post-hoc *p* = 0.93). With regards to latency variability, Levene’s test found that it was higher in SlowP vs. H children (F_1,18_ = 5.27, *p* = 0.034), while the difference between NonP and H did not reach significance (F_1,18_ = 3.48, *p* = 0.08).

### 3.4. Index Finger Kinematics Parameters

Figure 3 shows that movement latency (B) and amplitude (D) were comparable in the three groups (ANOVA F_2,27_ < 1.9, *p* > 0.17) while its duration (C) was about 50% longer (240 ms vs. 176 ms or 181 ms) in SlowP children (ANOVA F_2,27_ = 3.7, *p* = 0.038).

## 4. Discussion

### 4.1. Intra-Limb APAs in SlowP and NonP Children: Role of Disease Progression

Our data showed worse upper-limb postural control in SlowP than in NonP children. This result replicates the observation of Farinelli et al. [23] on quiet stance and gait initiation. As explained in that paper, such different behaviors may derive from the progressivity of the pathology. This idea stems from a series of observations about the motor behavior in subjects suffering from cerebellar agenesis, i.e., a loss of about 85 billion neurons [27], which should produce a catastrophe in motor behavior (see also Lemon and Edgley [28]). After the initial report by Combettes [29], the literature describes a few other cases of complete (or almost complete) cerebellar agenesis, in which patients showed a “mild or moderate” motor impairment [30,31,32,33,34,35]. In any case, these patients displayed a much less severe motor impairment than that usually seen after acute cerebellar damage. Many of the above papers ascribed such a result to the ability of intact extracerebellar motor structures to build up a functional compensation in the framework of a stable lesion since birth. In this regard, Arrigoni et al. [32] underlined that the severe neuropsychomotor deficit observed during infancy and adolescence in their “NonP” patients actually improved over the years. Seemingly, the framework of a stable lesion may represent a key aspect for creating and tailoring an effective functional compensation. In this perspective, the disease progressivity, such as that affecting our SlowP children, likely interfered with the consolidation of compensatory functional strategies, thus worsening the upper-limb postural control. Moreover, it seems quite unfeasible that the slight difference in duration of physical treatment between the two groups (on average 12.1 years in NonP and 10.1 years in SlowP; see methods) may have contributed to the clear difference in postural performance, considering that both groups should have already reached the maximal benefit well before 10 years of rehabilitation.

It is worth noting that the literature concerning cerebellar patients presents contrasting reports about the effect of a damaged cerebellum under APA control. Indeed, according to Mummel et al. [36], cerebellar vascular lesions do not preclude the APA adaptation to the mechanical context, while Timmann and Horak [16] reported that patients with chronic cerebellar degeneration displayed APAs preserved in timing. Instead, Muller and Dichgans [37] and Babin-Ratté et al. [38] reported that patients with cerebellar degenerations failed to show a normal anticipatory adjustment in grip force when lifting or moving an object. Finally, following the “common motor command identification” approach developed by the Latash group (M-modes [39,40,41,42]), Asaka and Wang [15] found that ataxic patients suffering from spinocerebellar degeneration showed altered feed-forward muscle synergies and multi-mode coordination when compared to healthy subjects. Taking into account our hypothesis, such contrasting reports may actually be a result of different degrees of progressivity of cerebellar diseases.

With regard to the functional repercussions of altered APAs, it is worth recalling that both the absence and the delay of APAs are linked to deficits in movement precision. Delayed APAs in the upper-limb worsen the precision of hand pointing movements [43], and the same was demonstrated for lacking APAs in a model of index finger flexion movements [14]. The linkage between altered APA amplitude and lack of precision has also been demonstrated in leg muscles during arm pointing movements [44].

### 4.2. Intra-Limb APAs in NonP and H Children: Putative Compensatory Mechanisms

We observed that APA timing and pattern in NonP children were not statistically distinguishable from those in H children, indicating that in these patients an efficient compensation developed, likely thanks to the lesion’s stability since birth, as in the many cases reported above. When debating about the possible compensation mechanisms, one should take into account the compensatory role of the cerebellum in early-onset parkinsonian patients reported by Wu and Hallett [45] and the existence of the cerebellum to basal ganglia connection, demonstrated in animal models [46]. The view of a highly integrated network between the basal ganglia, cerebellum and cortex has also been analyzed in detail in the consensus paper by Caligiore and colleagues [47]. These findings led to the idea that an intact basal ganglia may reciprocally compensate for cerebellar deficits [48]. This hypothesis is now further supported by preliminary tractography data showing a higher integrity of bidirectional connections between the basal ganglia and cerebellum in NonP children than in SlowP children [49]. In order to complete the puzzle, it would also be interesting to evaluate whether adults suffering from non-progressive cerebellar ataxia display a quasi-normal APA organization.

### 4.3. Intra-Limb APAs in Children and Adults: Role of Cerebellum in APA Ontogenesis

Another aspect that deserves a comment derives from the analogy between the delayed and more dispersed timing of APAs observed in SlowP children and that observed in “SlowP” adults [22]. The apparent delay in APA onset found in both populations recalls the dyschronometria of voluntary movement (a clinical hallmark of cerebellar ataxia) and provides further support to the idea that the activities in the prime mover and associated postural muscles are driven by a shared motor program [5,50]. It is also worth noting that the behavior we observed in H children was comparable in pattern and latency to that reported in healthy adults [5]. Confirming the early findings of Forssberg et al. [51], the development of APAs in childhood follows the time course described by Schmitz et al. [52] and reaches full maturity around the end of the first decade. It is therefore not surprising that cerebellar damage may cause severe alterations in postural control even at this age.

### 4.4. Are Inhibitory APAs Really Missing in Cerebellar Patients?

As reported in the results section, inhibitory APAs could not be detected in a large number of SlowP children (eight out of ten for AD and six for BB). This behavior matches that reported in “SlowP” adults by Bruttini et al. [22], despite being less frequent (four out of thirteen for both AD and BB). The question then arises as to whether to state that APAs were missing or that they were masked. In fact, both our and Bruttini’s work reported that all patients displayed the excitatory APA in TB but were consistently delayed when compared to healthy participants. Were it also true for inhibitory APAs, one could imagine that they were not missing but slipped under and were masked by the rebound excitation that occurs after movement onset. With regard to this last action, it cannot be said how much of it is pre-programmed or dependent on the reflex response triggered by kinesthetic reafferences. However, considering that cerebellar damage should have little effect on reflex responses and that the rebound excitation was present both in healthy and cerebellar subjects, the “reflex” interpretation seems to be more probable.

### 4.5. May APA Timing Be Affected by Bradykinesia?

Several papers reported that APAs may be scaled in latency according to the speed of the motor action [53,54,55]. Therefore, one could wonder if the delay observed in SlowP children might stem from their bradykinesia (Figure 3C). Note however that in the above papers the linkage between APAs and movement velocity was highlighted within single subjects, who were asked to voluntarily change their movement speed. Instead, the finger flexion paradigm always required that participants flex their index finger at maximal speed. It has been reported that the maximal flexion speed in the population of healthy adults covered a rather wide range (from about 250 to 1400°/s), but no significant correlation has been found between APA timing and movement speed [56]. This observation should rule out bradykinesia as a confounding factor based on the present data.

### 4.6. Limitations and Methodological Considerations

Despite its simplicity, both in terms of movement execution and required instrumentation, the index finger flexion paradigm seems to be very sensitive to detect APA deficits, in particular with regard to the pattern [57] and timing [22]. However, the main limitation of this experimental paradigm regards the quantification of APA amplitude, which has not been considered in this paper because the EMG signals in the presence of neuromuscular dysfunctions are often not comparable to those of healthy individuals. In this regard, two further issues should be taken into account.

One is the need for normalizing the EMG amplitude to a common reference value. This is usually performed by referencing the APA amplitude to the maximal voluntary contraction of the same muscle. However, this last measurement is likely to be misleading in the presence of motor deficits, which may differently affect anticipatory “phasic” activity vs. the voluntary “sustained” contraction in postural muscles. The other issue is the time window applied to amplitude measurements. The APA amplitude should be measured from its own onset up to the onset of the voluntary movement. In fact, beyond that time point, one cannot rule out that reflex components, due to kinesthetic afferences, mix up with the feedforward anticipatory action. However, such measurements would also be affected by changes in APA latency. For instance, APAs in SlowP children occurred later than in H (Figure 3A) despite a comparable movement latency (Figure 3B); this actually shortened the time window. Comparing APA amplitudes measured with different time windows produces inconclusive or even incorrect results. Indeed, such a measurement would reflect the correct APA amplitude only if the time window covered its full time course. If the time window ended before the APA peak, the measurement would approximate the slope (rate of change) of the APA. If instead the time window included the APA peak but not its end, the measurement would mix up amplitude and slope in variable proportions from case to case. Thus, one might choose a time window of fixed duration, which however could extend well beyond the movement onset, especially in SlowP children, again leading to a mix-up but in this case between feedforward and reflex components.

Briefly commenting on sample size and selection, the patients’ recruitment was on a voluntary basis, and the two kinds of Pediatric Cerebellar Ataxias (SlowP and NonP) are very rare pathologies. Moreover, patients had to be able to understand and perform the required motor task while actively keeping their upper limb in the required position. This obviously complicated the recruitment, limiting it to a subpopulation of SlowP and NonP children and making each group a “convenience” sample. However, at the same time, the above inclusion criteria also granted a sort of homogenization of the clinical pictures within each group. We have no reason to excuse the fact that recruitment within each group was not random. Finally, for what concerns the very young adult included in the NonP group, his performance was well within the range of NonP, his APA latencies being −23 ms for BB, +5 for TB and −35 for AD.

## 5. Conclusions

The present results support the suggestion that the progressivity of cerebellar disease may be a key factor in determining the impairment in postural control, actually endorsing it as a general principle that encompasses both *inter-* and *intra-limb* APAs. Furthermore, the observation that upper-limb postural control in the NonP group was not significantly worse than that of the H children highlights that only a fully developed compensatory network, unimpeded by the disease progression, may efficiently compensate for the impaired cerebellar functions. Taken together, such data may have an impact on the planning and design strategies of rehabilitative interventions for ataxic patients. The definition of the critical differences between non-progressive and slowly progressive ataxia could guide a personalized rehabilitation approach, maximizing the positive results deriving from therapy. Finally, the present paper confirms the role of the cerebellum in APA ontogenesis.

## Data Availability

Data presented in this study are available on reasonable request from the corresponding author.
